# Moisture-mediated resource availability shapes rhizosphere and bulk soil microbial structure and function post-rainfall

**DOI:** 10.3389/fmicb.2026.1752099

**Published:** 2026-06-24

**Authors:** Hengsheng Wang, Yanli Han, Chao Chen, Kelong Chen, Yan Zhang, Zhanjun Wang, Lulu Qi

**Affiliations:** 1 School of Biological and Food Engineering, Hefei Normal University, Hefei, China; 2 College of Geographic Sciences, Qinghai Normal University, Xining, China; 3 College of Ecological Environment and Resources, Qinghai Minzu University, Xining, China; 4 Industrial Crop Institute, Anhui Academy of Agricultural Sciences, Hefei, China

**Keywords:** *Poa alpigena*, metagenomics, microbial functional shift, niche-dependent changes, rainfall pulses

## Abstract

**Introduction:**

Rainfall pulses drive rapid ecological changes in alpine grasslands, but their compartment-specific effects on short-term soil microbial dynamics remain unclear.

**Methods:**

We investigated the structural and functional responses of rhizosphere versus bulk soil microbiomes associated with *Poa alpigena* in the Qinghai Lake Basin. Paired soil samples were collected before rainfall and 2 h after a heavy rainfall event and analyzed by shotgun metagenomic DNA sequencing.

**Results:**

Rainfall triggered compartment-specific shifts in microbial community assembly. In the rhizosphere, rainfall significantly reduced alpha diversity (Chao1 and Richness indices) but enhanced community evenness (Simpson and Shannon indices), whereas bulk soil diversity remained relatively stable. DNA-based functional profiling revealed a short-term shift in the rhizosphere from a pre-rain “carbon-oriented” metabolic potential to increased relative abundance of genes involved in central carbon pathways, amino acid degradation, and chemotaxis post-rainfall. Notably, sequences affiliated with *Paraburkholderia* were significantly enriched in the nitrogen-limited rhizosphere immediately after rainfall, suggesting a potential link to nitrogen cycling. In contrast, bulk soil communities shifted toward gene categories for labile carbon utilization and bacterial secretion systems. Co-occurrence network analysis indicated that rainfall simplified microbial interactions and weakened the coupling between microbial communities and soil physicochemical properties.

**Discussion:**

These findings demonstrate that rainfall pulses trigger rapid, niche-dependent changes in soil microbiomes at the DNA level, driven by moisture-mediated shifts in resource availability, and highlight distinct ecological strategies in rhizosphere and bulk soil compartments.

## 1 Introduction

Situated on the northeastern margin of the Qinghai–Tibet Plateau, the Qinghai Lake Basin is an ecologically critical transition zone with alpine wetland ecosystems that perform essential barrier functions, including water conservation, soil erosion control, and biodiversity maintenance. The basin harbors a unique and vulnerable ecosystem structure shaped by high-altitude conditions (average elevation > 3,000 m) and a semi-arid climate (annual precipitation 300–400 mm) (Hou et al., 2023; Yang et al., 2021). Ecological processes within the basin are governed by spatiotemporal patterns of precipitation, which directly modulate rhizosphere moisture dynamics and drive corresponding microbial responses (Wan et al., 2024). For instance, previous studies in alpine grasslands have shown that precipitation variability can substantially alter soil water potential over short periods, thereby directly affecting microbial metabolic activity (Liu et al., 2023). Concurrently, precipitation variability influences plant community biomass and cover; for example, a 30% increase in precipitation can enhance aboveground biomass of alpine forages by 15%–25%, thereby indirectly altering soil microbial composition through changes in root exudation rates (Niu and Fu, 2024).

The root–soil interface (rhizosphere) represents a key ecological niche wherein rhizosphere microbial communities facilitate plant–soil feedbacks via mechanisms such as nutrient solubilization, biological nitrogen fixation, and metabolic exchange—processes that collectively enhance plant nutrient uptake and resistance to environmental stressors (Trivedi et al., 2020). These microbial assemblages exhibit substantial heterogeneity in composition and diversity due to interspecific variation in root exudation patterns and root system morphology (Smilauer et al., 2020). For instance, graminaceous plants typically exude more organic acids than legumes, shaping distinct rhizosphere microbial communities dominated by chemoheterotrophic bacteria (Hu et al., 2018). Acting as dynamic bio-intermediaries, rhizosphere microbes regulate nutrient cycling, water utilization, and stress adaptation, thereby mediating ecological responses to precipitation pulses in semi-arid alpine regions (Wan et al., 2024; Yang et al., 2021).

*Poa alpigena* Lindm is recognized as a typical model forage species in alpine regions owing to its pronounced adaptability to high-altitude environments, vigorous regenerative ability, and superior forage characteristics (Fan et al., 2018). Its prostrate growth form and extensive root architecture confer resistance to low temperature, intense radiation, and abbreviated growing seasons, making it ideal for studying stress physiology and ecological adaptations in forage grasses. Additionally, *P. alpigena* supports alpine pastoral system stability through high nutritional content (crude protein 12%–15%) and exceptional grazing tolerance, serving as a key species for sustainable grassland productivity and ecological restoration (Fan et al., 2018). Notably, precipitation events trigger rapid physicochemical shifts in the rhizosphere microenvironment of this plant; For example, soil moisture content shows a marked increase shortly following precipitation events, resulting in a substantial and swift reorganization of the structural and functional characteristics of microbial communities (Brokate et al., 2024). This view is strongly supported by research in similar ecosystems. For instance, a study on Tibetan alpine grasslands demonstrated that water availability exerts a stronger influence than plant species identity in shaping microbial functional gene profiles, explaining a substantial portion of the variation in functional gene composition (Liu et al., 2023). Specifically, increased precipitation was found to be associated with an increased relative abundance of key microbial functional genes, particularly those involved in carbon and nitrogen cycling. Such dynamic microbial responses play a direct role in modulating the plant’s water-use efficiency and overall adaptability to environmental fluctuations (Heffernan and Fisher, 2012; Iqbal et al., 2025), and understanding this process is essential for elucidating the mechanisms underpinning the restoration of alpine sandy ecosystems. However, most existing studies, including the aforementioned work, have focused on the overall microbial responses to precipitation changes at the bulk soil level, with limited attention paid to the differential responses between the rhizosphere and bulk soil compartments (Liu et al., 2021). Given that rhizosphere and bulk soil differ in resource availability and microenvironmental conditions, their microbial communities likely exhibit distinct response patterns to rainfall pulses (Trivedi et al., 2020).

High-throughput metagenomic sequencing enables direct extraction and sequencing of total DNA from environmental samples, overcoming culture-based constraints that capture less than 1% of soil microbes (Parmar et al., 2024). This approach provides comprehensive profiling of microbial community structure, phylogenetic diversity, and functional potential at an ecosystem-wide level, accessing the vast reservoir of uncultivated microbial diversity—often termed “microbial dark matter”—and has catalyzed paradigm-shifting developments in environmental microbiology (Masuda et al., 2024; Trivett et al., 2025). For example, metagenomic studies have identified novel nitrogen-fixing bacteria in alpine soils that were previously undetected by culture-based methods, expanding our understanding of nitrogen cycling in high-altitude ecosystems (Shen et al., 2021).

The Qinghai Lake Sanjiaocheng Wetland is an alpine grassland agricultural ecosystem of considerable ecological and economic importance, characterized by an arid climate with limited rainfall (annual precipitation ∼322.7 mm) and high evaporation (∼1,500 mm), where precipitation events drive soil water dynamics and nutrient cycling (Hou et al., 2023). Investigating pre- and post-precipitation dynamics of rhizosphere microbial communities associated with superior forage species is essential for elucidating soil–plant–microbe–moisture interactions in high-altitude environments. This study employs shotgun metagenomics to characterize microbial functional gene repertoire shifts and assess their ecological implications for nutrient cycling. By integrating soil physicochemical data with microbial community profiles, we identify key environmental drivers of assemblage dynamics following water input, thereby deciphering plant-microbe co-adaptation mechanisms to alpine moisture fluctuations. It should be noted that this study adopts a “snapshot comparison” design with two static sampling time points (before rainfall and 2 h after rainfall cessation), which limits interpretation of continuous microbial dynamics. Seasonal variations in climate, plant phenology, and soil properties may lead to divergent response patterns (Niu and Fu, 2024; Yang et al., 2021). Therefore, the observed shifts should be interpreted as temporal associations rather than definitive causal relationships, and future multi-seasonal time-series studies are necessary to resolve the kinetics of microbial community reassembly.

## 2 Materials and methods

### 2.1 Study area

This research was conducted in the Sanjiaocheng Wetland (37°20’6″N, 100°14’43.4″E) along the northern shore of Qinghai Lake, at an elevation ranging from 3,200 to 3,300 m. The area exhibits a characteristic plateau semi-arid alpine climate, with a mean annual temperature of −0.7 °C (range: −31 °C to 28 °C) and annual precipitation averaging 322.7 mm. The landscape consists of typical alpine grassland, characterized by higher elevations in the northeast gradually descending to lower terrain in the southwest, flanked by mountainous formations along the northeastern margins. The dominant vegetation comprises species such as *Poa alpigena* L., *Stipa purpurea* Griseb., *Carex rigescens*, *Leymus secalinus*, *Polygonum sibiricum* L., *Allium przewalskianum*, and *Astragalus adsurgens* Pall., which collectively form a distinctive alpine wetland plant community.

### 2.2 Samples method and treatment

The sampling campaign was carried out in the Sanjiaocheng Wetland. Within four 2 m × 2 m quadrats exhibiting uniform coverage of *Poa alpigena* L., soil specimens were systematically acquired via an S-shaped five-point sampling strategy. Each quadrat served as one biological replicate, resulting in 4 biological replicates for each of the four sample groups (SJC: pre-rainfall bulk soil; SJG: pre-rainfall rhizosphere soil; YSJC: post-rainfall bulk soil; YSJG: post-rainfall rhizosphere soil). To clearly distinguish between rhizosphere and bulk soil compartments, two distinct sampling protocols were employed. For rhizosphere soil (designated as SJG and YSJG), whole plants with intact root systems were carefully excavated using a sterile spade. The aboveground plant material was removed, and the root systems were placed in sterile plastic bags for transport. In the laboratory, roots with tightly adhering soil were first gently shaken for 30 s to remove loosely attached soil, which was discarded. The remaining soil firmly adhering to the root surface was operationally defined as rhizosphere soil. To collect this fraction, roots were transferred to sterile 50 mL centrifuge tubes containing 25 mL of sterile phosphate-buffered saline (PBS, pH 7.4). The tubes were vortexed at 3,000 rpm for 2 min to dislodge the rhizosphere soil. After vortexing, the roots were aseptically removed with sterile forceps, and the resulting soil suspension was centrifuged at 10,000 × *g* for 10 min at 4 °C to pellet the rhizosphere soil. The supernatant was discarded, and the soil pellet was collected as the rhizosphere sample. For bulk soil (designated as SJC and YSJC), samples were collected from bare areas devoid of any vegetation, located at least 20 cm away from the nearest *P. alpigena* plants to ensure complete exclusion of root influence. Prior to sampling, the absence of visible plant roots was confirmed by visual inspection of the soil surface and a preliminary assessment of the top 2 cm layer. Soil was collected from the adjacent 0–20 cm surface layer using a sterile soil auger. After collection, the soil was immediately homogenized by passing through a 2 mm sterile sieve to remove stones, large organic debris, and any fine roots that may have been inadvertently collected, ensuring that the final bulk soil sample was free of root material and represented the non-rhizosphere soil compartment. Post-rainfall rhizosphere (YSJG) and bulk soils (YSJC) were sampled following an identical protocol. Meteorological records from the regional hydrological bureau indicated that the Qinghai Lake basin experienced a heavy rainfall event during the sampling night—as per the classification in the Chinese National Standard “Grade of Precipitation” (GB/T 28592-2012), with 12-h accumulations ranging from 15.0 to 29.9 mm. Post-precipitation sampling was initiated exactly 2 h after rainfall cessation. Both rhizosphere and bulk soil samples were subdivided into two portions. The first subset was immediately flash-frozen in liquid nitrogen for genomic DNA extraction and subsequent metagenomic sequencing. Samples were fully submerged in liquid nitrogen for a minimum of 5 min to ensure complete and rapid freezing. After flash-freezing, the samples were immediately transferred to a −80 °C freezer and stored continuously at this temperature until DNA extraction was performed, to prevent nucleic acid degradation. After extraction, precipitation, and purification, high-quality DNA preparations were submitted to BGI Technology Co., LTD (Shenzhen, China) for sequencing on the BGI-SEQ-500 platform. The second subset was allocated for physicochemical characterization, encompassing measurements of moisture content, total nitrogen (TN), total carbon (TC), pH, and electrical conductivity (EC). Analytical methodologies and instrumentation were applied as follows: soil water content was assessed with a JK-100F soil moisture analyzer (Shanghai Jingke Industrial Co., Ltd., China); Total nitrogen was quantified using a FOSS Kjeltec 8400 automated Kjeldahl unit (FOSS Analytical, Denmark); Total carbon was analyzed with a CE-440 elemental analyzer (Exeter Analytical Inc., USA); pH was measured employing a pHS-25 laboratory pH meter (Shanghai Leici Instrument Co., China); and electrical conductivity was determined via a DDS-307 conductivity meter (Shanghai Yidian Scientific Instrument Co., Ltd., China).

### 2.3 DNA extraction and metagenomic sequencing

Total genomic DNA was isolated from soil samples following the established protocol of Han et al. (2010). The extraction procedure involved the use of chloroform-isoamyl alcohol, followed by isopropanol reprecipitation and purification with QIAquick Gel Extraction Kit buffer. After quantification and quality assessment of the DNA, we employed a combination of three methods to ensure the reliability of DNA quality and quantity for subsequent metagenomic sequencing: spectrophotometer (Thermo Scientific, United States) for the initial assessment of nucleic acid purity (A260/A280 and A260/A230 ratios), a Qubit^®^ 4 Fluorometer (Invitrogen, United States) for precise quantification of DNA concentration, and 1% agarose gel electrophoresis for the visualization of DNA integrity and the detection of potential degradation or contamination. Metagenomic libraries were constructed using the NEBNext^®^ Ultra™ DNA Library Prep Kit for Illumina^®^ (NEB, United States) according to the manufacturer’s instructions. Briefly, 300 ng of high-quality genomic DNA (A260/A280 = 1.8–2.0, A260/A230 ≥ 1.5) was sheared into fragments of approximately 350 bp using a Covaris M220 ultrasonicator (Covaris, United States). The fragmented DNA was then subjected to end repair, A-tailing, and adaptor ligation. After adaptor ligation, DNA fragments were purified and size-selected using AMPure XP beads (Beckman Coulter, United States) to retain fragments of 300–400 bp (average library size: 350 bp). The libraries were quantified using a Qubit^®^ 4 Fluorometer (Invitrogen, United States) to ensure a final concentration of 10 nM, and library quality was verified by 1% agarose gel electrophoresis and Agilent 2100 Bioanalyzer (Agilent Technologies, United States) before sequencing. Metagenomic libraries were constructed and subjected to high-throughput sequencing on the DNBSEQ™ platform at BGI (Shenzhen, China).

### 2.4 Data analysis

Raw sequencing reads were trimmed to remove adapter sequences and low-quality bases using Trimmomatic v0.39 with the parameters SLIDINGWINDOW:4:20 and MINLEN:50 (Bolger et al., 2014). Specifically, the quality score threshold was set to Q20, meaning that bases with a Phred quality score < 20 were considered low-quality and trimmed; in addition, leading and trailing low-quality bases (below Q20) were removed using the LEADING:20 and TRAILING:20 parameters, respectively. Clean reads were subjected to *de novo* metagenomic assembly using MEGAHIT v1.2.9 with k-mer sizes ranging from 21 to 141 bp (in increments of 10 bp), which is a widely used k-mer setting for soil metagenomic assembly to balance assembly continuity and accuracy (Li et al., 2016). The quality of assembled contigs was evaluated using MetaQUAST v5.2.0, including alignment statistics, contig length distribution, and N50 values (Mikheenko et al., 2016).

Taxonomic annotation was performed using Kraken2 against the NCBI RefSeq complete genome database, including bacterial, archaeal, fungal, protozoan, and viral complete genomes, plus the NCBI NT database. The similarity threshold for taxonomic classification was set according to the default parameters recommended by the sequencing facility for soil metagenomic profiling, consistent with standard Kraken2 practices (Wood et al., 2019). Subsequent species-level abundance estimation was refined using Bracken v2.0 (Lu et al., 2017).

Microbial α-diversity indices were calculated using the Vegan package v2.6-4 in R environment (Oksanen et al., 2022). Comparisons of alpha diversity indices (Chao1, Richness, Simpson, and Shannon) among the four sample groups (SJC, SJG, YSJC, YSJG) were performed using One-way analysis of variance (One-way ANOVA) followed by Tukey’s Honestly Significant Difference (HSD) *post-hoc* test for multiple comparisons, which is a standard statistical approach for alpha diversity comparison in soil microbial metagenomic studies (Liu et al., 2023; Niu and Fu, 2024). All *p*-values were adjusted for multiple comparisons using the Tukey HSD method, and statistical significance was defined as adjusted *p* < 0.05. All *p*-values reported for alpha diversity comparisons were derived from this statistical method. For β-diversity analysis, permutational multivariate analysis of variance (PERMANOVA) was performed using the adonis2 function in the Vegan package v2.6-4 with 999 permutations to test for significant differences in microbial community composition among sample groups. The Bray-Curtis dissimilarity matrix was used as the distance metric, and significance was defined as *p* < 0.05. β-diversity was visualized using principal component analysis (PCA) based on normalized taxonomic abundance profiles. To identify differentially abundant taxa and functional features, Linear Discriminant Analysis Effect Size (LEfSe) was applied. First, the non-parametric factorial Kruskal-Wallis test was used to detect significant differences among groups with a significance threshold of α = 0.05. Then, the Wilcoxon rank-sum test was performed between pairwise subgroups (also at α = 0.05) to confirm consistent differences. Finally, an LDA score threshold > 2.0 was used to select features with the greatest effect size (Segata et al., 2011). Microbial metabolic pathway enrichment analysis was performed following the pipeline described by Zorrilla et al. (2021).

For functional annotation and KEGG pathway enrichment analysis, the HUMAnN3 v3.6.1 pipeline was employed (Beghini et al., 2021). Briefly, translated reads were aligned to the KEGG database (Release 104.0) using a translated search algorithm. Abundances of KEGG pathways were normalized to copies per million (CPM) to account for differences in sequencing depth. Pathway enrichment was determined using a false discovery rate (FDR) threshold of <0.05 and a fold-change threshold of ≥1.5 to identify significantly enriched metabolic pathways.

All statistical analyses were performed using SPSS v26.0 (IBM Corp., Armonk, NY, United States) and R v4.2.0 (R Core Team, 2022). For alpha diversity comparisons, One-way ANOVA with Tukey’s HSD *post-hoc* test was used (*p*-Values < 0.05 was considered statistically significant). Raw sequencing data were deposited in the NCBI Sequence Read Archive (SRA) under BioProject accessions PRJNA867494 and PRJNA1188057, with the following run accessions:

SJC group: SRX17159234–SRX17159237

SJG group: SRX17159238–SRX17159241

YSJC group: SRX26957323, SRX26957324, SRX26957326, SRX26957327

YSJG group: SRX26957319–SRX26957322

## 3 Results

### 3.1 Analysis of soil physicochemical properties in the rhizosphere and bulk soil of *Poa alpigena* before and after a rainfall event in the Sanjiaocheng sample plot

A comparative analysis of the physicochemical properties was conducted on soil samples collected from the rhizosphere and bulk soil zones of *P. alpigena* in the Sanjiaocheng sample plot, both prior to and following a rainfall event (Table 1). The results revealed considerable variations in soil moisture content across the four sampling categories, with mean values ranging from 4.2% to 13.78%. Pre-rainfall, the moisture content in the rhizosphere soil was significantly greater than that in the bulk soil (*p*-value < 0.05). In contrast, post-rainfall, this differential was no longer statistically significant, indicating an observed homogenization pattern that coincided with precipitation. The average soil pH value exhibited a range between 7.83 and 9.13 across all samples. A significant decrease in pH was observed in the bulk soil after rainfall (*p*-value < 0.05). Conversely, the pH of the rhizosphere soil demonstrated remarkable stability, showing no significant change. This disparity suggests an enhanced buffering capacity within the rhizosphere microenvironment, which likely confers a greater resilience to environmental fluctuations, shaped by rainfall. Electrical conductivity (EC), a key indicator of soluble salt content, showed average values ranging from 0.1 to 0.49 mS/cm. Post-rainfall, a significant increase in EC was detected in both the rhizosphere and bulk soils (*p*-value < 0.05). Notably, the magnitude of this increase was significantly more pronounced in the rhizosphere compared to the bulk soil. This indicates a potentially stronger adsorption or retention of solutes within the root zone, possibly due to physicochemical interactions between soil minerals, organic matter, and root exudates. The mean concentrations of total nitrogen (TN) and total carbon (TC) ranged from 0.24% to 0.49% and from 3.26% to 5.03%, respectively. Prior to the rainfall event, the concentrations of both total C and N were significantly enriched in the rhizosphere soil compared to the bulk soil (*p*-value < 0.05). Following rainfall, a significant reduction in TC and TN content was observed in the rhizosphere, while the bulk soil exhibited a significant increase (*p*-value < 0.05). This reciprocal shift suggests that rainfall may have facilitated the mobilization and subsequent translocation of nutrients from the nutrient-rich rhizosphere to the surrounding bulk soil, promoting a more homogeneous spatial distribution of nutrients across the soil matrix. However, this pattern could also be explained by alternative mechanisms, such as differential microbial immobilization in the two compartments or leaching of dissolved nutrients below the sampling depth, which warrant further investigation.

**TABLE 1:** Physicochemical properties of bulk and rhizosphere soils across different sampling sites.

Site name	H_2_O (%)	pH	EC (mS/cm)	TC (%)	TN (%)
SJC	4.2 ± 0.82^b^	9.13 ± 0.25^a^	0.26 ± 0.05^b^	3.26 ± 0.32^b^	0.24 ± 0.07^c^
SJG	7.17 ± 1.78^b^	8.21 ± 0.35^b^	0.10 ± 0.02^b^	5.03 ± 0.75^a^	0.49 ± 0.08^a^
YSJC	13.23 ± 1.72^a^	7.83 ± 0.24^b^	0.47 ± 0.09^a^	4.00 ± 0.16^b^	0.37 ± 0.05^b^
YSJG	13.78 ± 1.45^a^	8.19 ± 0.36^b^	0.49 ± 0.15^a^	3.87 ± 0.41^b^	0.39 ± 0.06^ab^

Values represent mean ± SD. Different superscript letters within a column denote significant differences (*p*-value < 0.05) between bulk/rhizosphere soils at different sites. TN, total nitrogen; EC, electrical conductivity; TC, total carbon.

### 3.2 Taxonomic composition and statistical overview of microbial communities

To characterize the taxonomic composition of soil microbial communities in the Sanjiaocheng region under varying moisture conditions, metagenomic sequencing was employed. The analysis revealed significant shifts in microbial community structure linked to precipitation and rhizosphere presence. Notably, the proportion of high-quality sequences assignable to microbial taxa differed markedly among conditions. Pre-rainfall samples were dominated by microbial sequences, comprising 39.77% and 41.16% in rhizosphere (SJG) and bulk soil (SJC) samples, respectively. In contrast, post-rainfall samples showed a substantial reduction, with microbial sequences accounting for only 25.80% (YSJG, rhizosphere) and 23.66% (YSJC, bulk soil). This drastic decline from ∼40% to ∼25% in microbial read proportions post-rainfall is likely attributed to a dilution effect, which is validated by the potential influx of plant-derived DNA into the soil matrix following precipitation. Rainfall can leach plant debris, root exudates, and fragmented plant tissues into the soil, increasing the relative abundance of plant DNA in the total extracted nucleic acids and thereby reducing the proportion of detectable microbial sequences without necessarily altering the absolute abundance of microbes. Bacteria constituted the dominant domain across all communities, yet their relative abundance varied significantly with environmental conditions. The highest bacterial abundance was observed in pre-rainfall bulk soil (SJC) samples (33.66%), which was significantly higher (*p*-value < 0.05) than in post-rainfall bulk soil (YSJC) samples (20.62%). Fungal sequences were consistently present at much lower abundances (0.40%–1.48%). Other groups, including protozoa and viruses, collectively represented less than 0.2% of classified sequences in all samples (Table 2), indicating their minimal contribution to the overall community under the studied conditions. It is important to note that the substantial reduction in the proportion of microbial reads post-rainfall is likely attributable to a “dilution effect” caused by increased input of plant-derived DNA into the soil matrix following precipitation. This dilution can alter the relative abundances of microbial taxa without necessarily reflecting changes in their absolute abundances. Therefore, all subsequent interpretations of relative abundance data (e.g., shifts in Proteobacteria) should be viewed as changes in relative proportions rather than definitive evidence of absolute biological shifts, and this confounding factor must be considered when drawing ecological inferences (Supplementary Table 1).

**TABLE 2:** Pre-/post-rainfall sequencing of rhizosphere and bulk soil microbiomes in alpine *Poa alpigena*.

Site name	Number of raw reads	Classified reads (%)	Microbial reads (%)	Viral reads (%)	Bacterial reads (%)	Fungal reads (%)	Protozoan reads (%)
SJC	37948306.00 ± 22206.65^a^	47.00 ± 1.71^a^	41.16 ± 1.76^a^	0.16 ± 0.00^a^	33.66 ± 1.73^a^	1.33 ± 0.04^b^	0.17 ± 0.00^b^
SJG	37359562.50 ± 722911.70^a^	46.13 ± 1.12^a^	39.77 ± 1.14^a^	0.16 ± 0.00^a^	31.88 ± 1.14^a^	1.48 ± 0.04^a^	0.18 ± 0.00^a^
YSJC	37164104.50 ± 1602036.39^a^	25.22 ± 1.32^b^	23.66 ± 1.37^b^	0.05 ± 0.00^b^	20.62 ± 1.33^b^	0.40 ± 0.02^c^	0.05 ± 0.00^c^
YSJG	38390602.00 ± 9100.61^a^	27.49 ± 0.15^b^	25.80 ± 0.11^b^	0.05 ± 0.00^b^	22.61 ± 0.21^b^	0.45 ± 0.02^c^	0.05 ± 0.00^c^

Percentages in the table represent the proportion of each sequence relative to the total sequences. Data are expressed as mean ± SD. Different superscript letters indicate statistically significant differences (*p*-value < 0.05) among different sample groups within the same category.

### 3.3 Variations in soil microbial abundance and alpha diversity in response to rainfall events

**FIGURE 1:**
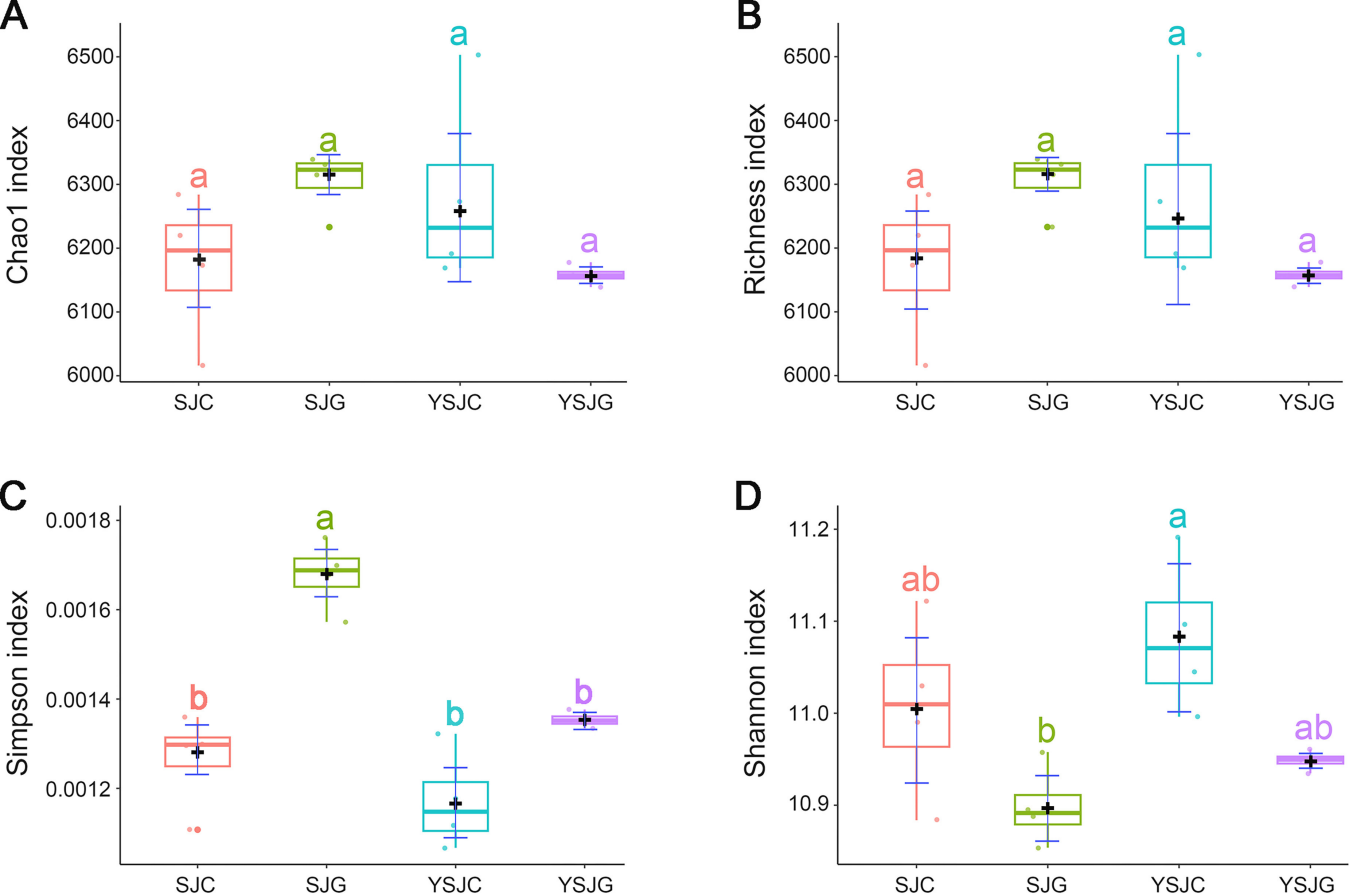
Soil microbial alpha diversity in the rhizosphere and bulk soil of *Poa alpigena* before and after rainfall. Boxplot showing different α-diversity indices. [**(A)** Chao1 index. **(B)** Richness index. **(C)** Simpson index. **(D)** Shannon index] for the four sample groups (SJC, SJG, YSJC, YSJG), Blue lines represent the standard deviation (SD), and the black “+” symbols within the boxes indicate the mean values. The letters a, b, and ab denote the results of pairwise comparisons among groups: different letters indicate statistically significant differences at *p* < 0.05, whereas the same letter indicates no significant difference.

Alpha diversity serves as a fundamental indicator for evaluating the composition and structural distribution of microbial communities. In this study, we assessed the alpha diversity of rhizospheric and non-rhizospheric soil microbiomes under pre-rainfall (SJC, SJG) and post-rainfall (YSJC, YSJG) conditions using four established indices: Chao1, Richness, Simpson, and Shannon. The results provide insights into the combined influences of rainfall and root proximity on microbial community diversity. As illustrated in Figure 1A, the Chao1 index, which estimates species richness, was significantly higher in the SJG (pre-rainfall rhizosphere) and YSJC (post-rainfall bulk soil) groups compared to the SJC and YSJG groups (*p*-value < 0.05), suggesting enhanced microbial richness in these niches. Similarly, the Richness index (Figure 1B), indicative of the total number of species, showed congruent patterns, with significantly elevated values in SJG and YSJC samples relative to SJC and YSJG (*p*-value < 0.05), thereby corroborating the Chao1 index findings. The Simpson index (Figure 1C), a measure of dominance concentration within the community, was significantly higher in the SJG group than in the SJC, YSJC, and YSJG groups (*p*-value < 0.05), reflecting greater diversity and reduced dominance in pre-rainfall rhizosphere soils. No statistically significant differences were detected among the latter three groups (*p*-value > 0.05). In contrast, the Shannon index (Figure 1D), which integrates both species richness and evenness, revealed significantly higher values in the YSJC group compared to the SJG group (*p*-value < 0.05), implying higher diversity and more uniform species distribution in post-rainfall bulk soils. No significant differences were observed between SJC and YSJG groups (*p*-value > 0.05). Collectively, these alpha diversity metrics highlight niche- and rainfall-driven shifts in microbial community structure, with pre-rainfall rhizosphere and post-rainfall bulk soils exhibiting distinctly enriched and diverse profiles.

### 3.4 Analysis of dominant soil microbial abundance before and after rainfall

**FIGURE 2:**
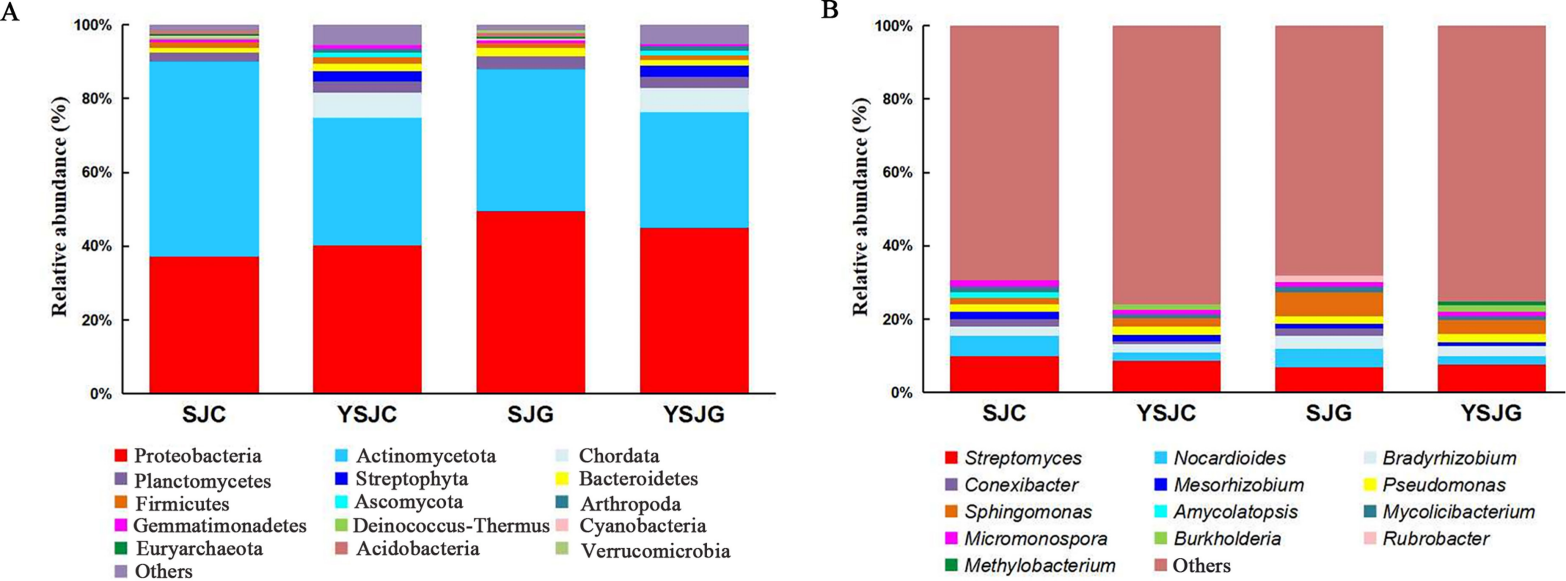
Soil microbial community composition of the four sample groups (SJC, SJG, YSJC, YSJG) at the phylum **(A)** and genus **(B)** levels.

Metagenomic sequencing and taxonomic analysis of genomic DNA were performed on rhizosphere and bulk soil samples of *P. alpigena* from the Sanjiaocheng region before and after rainfall (SJC, SJG, YSJC, YSJG). The results revealed that at the phylum level (Figure 2A), Proteobacteria and Actinomycetota were the absolutely dominant taxa, collectively accounting for over 75% of the total microbial community. Specifically, Proteobacteria represented between 37.2% and 49.5%, while Actinomycetota comprised 31.2%–52.8%, with these two phyla exhibiting inverse abundance patterns across different sample types, suggesting distinct ecological preferences or differential responses to environmental conditions rather than necessarily indicating direct competition. Further statistical analysis showed that the abundance of Proteobacteria was highest in pre-rainfall rhizosphere soil (SJG), significantly exceeding that in the other three sample types (*p*-value < 0.05). Moreover, its abundance was consistently higher in rhizosphere than in bulk soils. This distribution pattern may be attributed to the continuous accumulation of root exudates (e.g., organic acids, sugars) before rainfall, which could provide a metabolic advantage for chemoheterotrophic Proteobacteria. In contrast, Actinomycetota reached its highest abundance (52.8%) in pre-rainfall bulk soil (SJC), which was significantly greater than in all other samples (*p*-value < 0.05), and was generally higher before rainfall than after. This inverse abundance pattern between the two dominant phyla likely reflects their contrasting physiological adaptations and niche preferences—with Actinomycetota possessing drought-resistant spores well-suited to pre-rainfall dry conditions, while Proteobacteria respond rapidly to moisture-induced increases in labile carbon availability—rather than necessarily indicating direct competitive exclusion. At the genus level (Figure 2B), the top 10 most abundant microorganisms were predominantly Streptomyces, Nocardioides, and Bradyrhizobium, which together accounted for over 12% of the total sequences, identifying them as the dominant genera. Streptomyces exhibited its highest average abundance in pre-rainfall bulk soil, which was significantly greater than in the other three sample types (*p*-value < 0.05), with bulk soils overall showing higher levels than rhizosphere soils. The relative abundance of Nocardioides was significantly increased in both rhizosphere and bulk soils after rainfall, with no significant difference observed between the two post-rainfall groups (mean relative abundance: 2.50% in YSJC and 2.48% in YSJG). Meanwhile, these post-rainfall values were significantly higher than those in pre-rainfall bulk soil (SJC, 1.85%) and rhizosphere (SJG, 1.56%) soils.

### 3.5 Divergent responses of rhizosphere and bulk soil microbial communities to rainfall event revealed by LEfSe analysis

**FIGURE 3:**
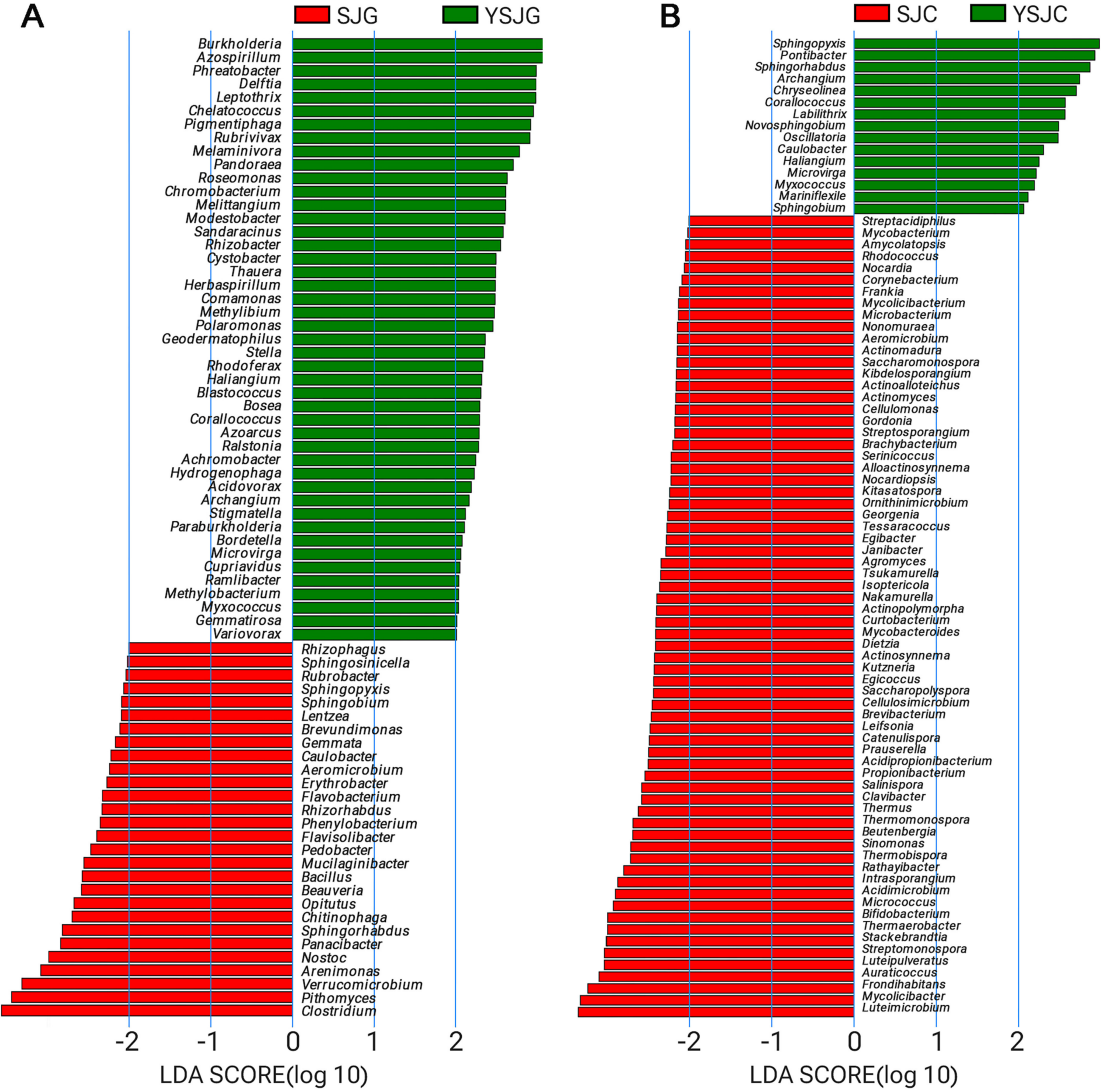
Differential soil microbes (genus level) between rhizosphere [**(A)** SJG–YSJG] and bulk soil [**(B)** SJC–YSJC] before and after rainfall.

Comparative linear discriminant analysis effect size (LEfSe) revealed that rainfall events and rhizosphere effects collectively was associated with the microbial community structure. In the rhizosphere soil, post-rainfall samples (YSJG) were significantly enriched in bacterial genera such as *Burkholderia*, *Azospirillum*, and *Pseudomonas*. In contrast, pre-rainfall rhizosphere samples (SJG) were dominated by *Sphingomonas*, *Paenibacillus*, and *Methylobacterium* (Figure 3A). Within the bulk soil compartment, genera including *Sphingopyxis*, *Pseudomonas*, and *Sphingobium* were significantly more abundant after rainfall (YSJC), whereas pre-rainfall samples (SJC) were characterized by a higher relative abundance of *Mesorhizobium*, *Micromonospora*, and *Agrobacterium* (Figure 3B). These results reveal significant temporal differences in microbial community composition, a pattern consistently observed in both rhizosphere and bulk soil. The enrichment of putative plant-growth promoting rhizobacteria (PGPR), such as *Burkholderia* and *Azospirillum*, in the pre-rainfall rhizosphere suggests their potential role in enhancing plant fitness through biological nitrogen fixation and phytohormone production. The shifts in the rhizosphere community following rainfall are likely associated with increased soil moisture and nutrient mobilization, which may selectively stimulate copiotrophic bacterial groups. Conversely, the bulk soil community response appeared to be driven predominantly by rainfall-induced changes in physicochemical properties, including variations in water potential and dissolved organic matter availability. Taxa that became enriched in post-rainfall bulk soil may possess adaptive traits that confer a competitive advantage under transiently elevated moisture and nutrient conditions.

### 3.6 Differential responses of soil microbial metabolism to rainfall and rhizosphere effects revealed by KEGG pathway analysis

**FIGURE 4:**
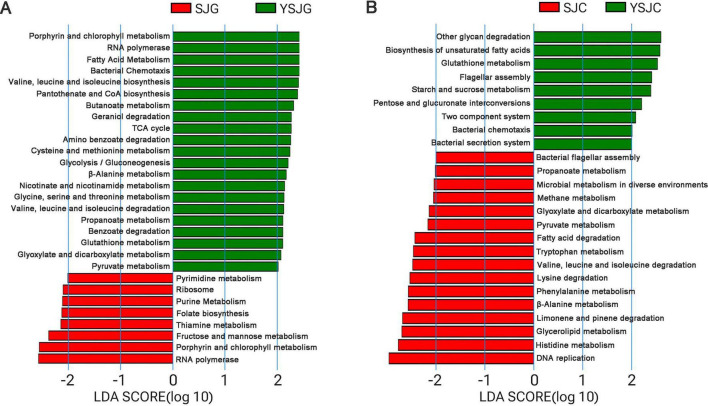
KEGG metabolic pathways of soil microorganisms in bulk soil (**(A)** SJC–YSJC) and rhizosphere soil [**(B)** SJG–YSJG] before and after rainfall.

Comparative analysis of KEGG metabolic pathways between pre- and post-rainfall rhizosphere (SJG vs. YSJG) and bulk soils (SJC vs. YSJC) demonstrated that rainfall significantly alters microbial functional profiles. In the rhizosphere (SJG vs. YSJG), post-rainfall samples (YSJG) exhibited significant enrichment of multiple metabolic pathways (LDA score > 2.0, *p*-value < 0.05; Figure 4A). These included pathways related to transcription and translation machinery (e.g., RNA polymerase), energy acquisition [Tricarboxylic acid (TCA) cycle, Glycolysis/gluconeogenesis, Pyruvate metabolism], and key biosynthetic processes (Fatty acid metabolism, various amino acid metabolisms such as Cysteine and Methionine metabolism, Glycine, Serine and Threonine metabolism). Additionally, YSJG soils showed significant enrichment of pathways associated with Chemotaxis-related functions, Butyrate metabolism, Benzoate degradation, and Glutathione metabolism (LDA score > 2.0, *p*-value < 0.05). In contrast, pre-rainfall rhizosphere samples (SJG) were characterized by the significant enrichment of a narrower set of pathways (LDA score > 2.0, *p*-value < 0.05), including Porphyrin metabolism, Branched-chain amino acid biosynthesis (Valine, leucine, and isoleucine biosynthesis), and cofactor synthesis (Pantothenate and CoA biosynthesis). Similar trends were observed in bulk soil (SJC vs. YSJC), where post-rainfall samples (YSJC) were significantly enriched in pathways related to energy metabolism, Fatty acid biosynthesis, redox homeostasis (Glutathione metabolism), motility (Flagellar assembly, Bacterial chemotaxis), carbohydrate metabolism (Starch and sucrose metabolism), and Protein secretion systems (LDA score > 2.0, *p*-value < 0.05; Figure 4B). These findings indicate that rainfall is associated with marked changes in microbial functional gene profiles, with more extensive shifts in gene relative abundance observed in the rhizosphere. This heightened response may be attributed to increased soil moisture, nutrient mobilization, and shifts in root exudate profiles following precipitation. The functional gene composition shifts observed in rhizosphere microbial communities underscore their adaptability to rapid environmental changes. In comparison, the more constrained functional shifts in bulk soil suggest either a buffered micro-environmental response or greater functional resilience to transient hydrological perturbations. Collectively, these results provide mechanistic insight into how rainfall modulates microbial metabolic potential, enhancing our understanding of microbially driven nutrient cycling—particularly carbon and nitrogen transformations—in soil ecosystems.

### 3.7 Analysis of soil microbial correlations and their relationship with soil physicochemical properties before and after rainfall events

**FIGURE 5:**
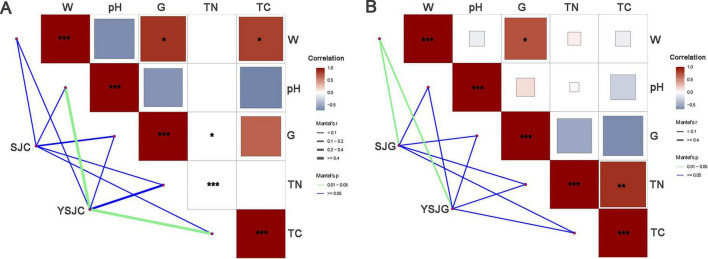
Correlation analysis between soil microbial communities and physicochemical properties in bulk soil [**(A)** (SJC–YSJC) and rhizosphere soil **(B)** (SJG–YSJG)] from Sanjiaocheng Wetland of Qinghai Lake before and after rainfall. Significance levels: *p < 0.05; **p < 0.01; ***p < 0.001.

To assess the relationship between overall microbial community composition and soil physicochemical variables, Mantel tests were performed using Bray–Curtis distances for microbial communities (SJG vs. YSJG and SJC vs. YSJC) and Euclidean distances for standardized physicochemical parameters (water content, pH, electrical conductivity, total nitrogen, and total carbon). The Mantel test was conducted with 999 permutations, which is the standard permutation number for Mantel tests in microbial ecology studies to ensure statistical reliability (Deng et al., 2012; Liu et al., 2021). The analysis revealed that within bulk soils, pre-rainfall microbial communities (SJC) showed no significant correlations with any physicochemical factors (*p*-value > 0.05). In contrast, post-rainfall bulk soil communities (YSJC) were significantly correlated with pH and total carbon (*p*-value < 0.05), but not with water content, electrical conductivity, or total nitrogen (*p*-value > 0.05) (Figure 5A). In the rhizosphere, both pre- and post-rainfall communities (SJG and YSJG) were significantly associated with soil water content (*p*-value < 0.05), but not with other physicochemical properties (Figure 5B).

**FIGURE 6:**
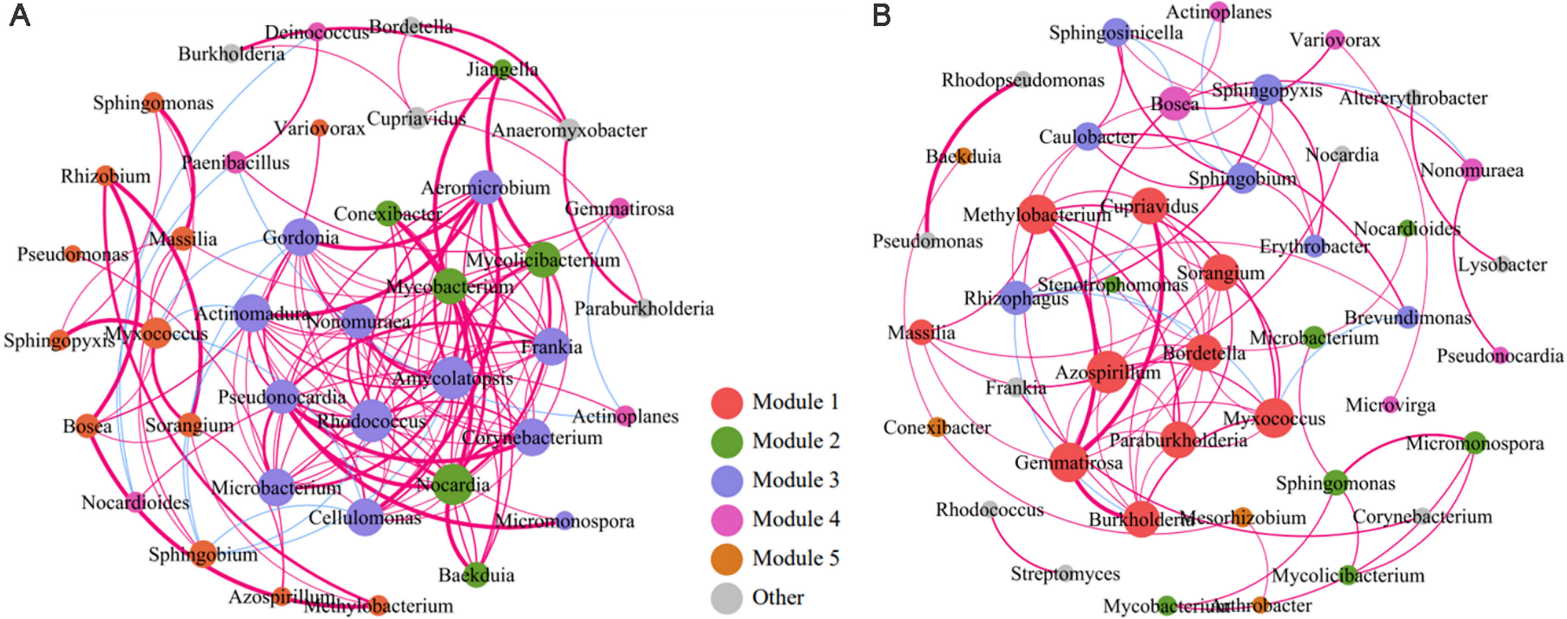
Inter-group correlation analysis of distinct soil microbial communities between bulk soil [**(A)** SJC–YSJC) and rhizosphere soil (**(B)** SJG–YSJG] from Sanjiaocheng Wetland of Qinghai Lake before and after rainfall.

To further elucidate how rainfall modulates microbial interkingdom interactions in rhizosphere and bulk soils, genus-level co-occurrence networks were constructed from microbial abundance data. These networks are constructed based on Spearman’s rank correlations of microbial relative abundances, which were calculated using the cor.test function in R v4.2.0 with the method = “spearman” parameter. To control for false positives caused by multiple testing and ensure the reliability of correlation results, the false discovery rate (FDR) correction was applied to the *p*-values using the Benjamini-Hochberg method (Benjamini and Hochberg, 1995), with a corrected *p*-value threshold of <0.05. Additionally, a Spearman correlation coefficient absolute value threshold of |r| > 0.4 was used to filter meaningful correlations, as this threshold is widely adopted in soil microbial co-occurrence network studies to balance network robustness and biological interpretability (Deng et al., 2012; Li et al., 2025), and thus reflect potential correlations rather than direct biological interactions. Additionally, the number of replicates in this study is limited, which may affect the stability and robustness of the constructed networks—this limitation is fully acknowledged and discussed in section “4.4 Methodological considerations and key limitations.” Networks were visualized in Gephi 0.9.7 (Bastian et al., 2009) using the ForceAtlas2 layout with default repulsion strength (1,000) and gravity (1.0). Edges were colored to indicate strong positive (red, r > 0.6) and strong negative (blue, r < −0.4) correlations. Comparative network analysis revealed marked structural differences between pre- and post-rainfall conditions in both rhizosphere and bulk soil communities (Table 1). For the bulk soil network (Figure 6A), we identified 40 nodes (genera) and 327 edges, with an average degree of 16.35, average path length of 2.12, and modularity of 0.58. This network included ecologically important taxa such as *Deinococcus*, *Bordetella*, *Burkholderia*, *Anaeromyxobacter*, *Rhizobium*, and *Paenibacillus*. Using the within-module connectivity (Zi) and among-module connectivity (Pi) criteria (Zi > 2.5, Pi > 0.62; Olesen et al., 2007), keystone taxa were identified as *Aeromicrobium*, *Mycobacterium*, *Nocardia*, and *Actinomadura*, which exhibited extensive positive correlations (r > 0.6, *p*-value < 0.05) with other genera. In contrast, *Sphingobium*, *Cellulomonas*, and *Gordonia* were primarily involved in negative correlations (r < −0.4, *p*-value < 0.05) with multiple partners, which may potentially indicate competitive associations—though this interpretation remains hypothetical due to the correlative nature of the data. These correlative patterns lead us to hypothesize that bulk soil microbial assemblages may enhance community stability through potential mutualistic correlations under resource-limited conditions. For the rhizosphere soil network (Figure 6B; He et al., 2023), 36 nodes and 189 edges were detected, with an average degree of 10.50, average path length of 2.78, and modularity of 0.63. Key taxa such as *Methylobacterium*, *Azospirillum*, *Cupriavidus*, and *Gemmatirosa* showed significant positive correlations (r > 0.6, *p*-value < 0.05) with other community members, whereas *Rhizophagus* and *Myxococcus* displayed limited but significant negative correlations (r < −0.4, *p*-value < 0.05). The rhizosphere network exhibited lower connectivity (Average degree = 10.50 vs. 16.35) and a sparser topology (Edges = 189 vs. 327) than the bulk soil network, which suggests weaker correlative patterns among microbes in the rhizosphere compared to bulk soil—rather than definitive differences in direct microbial interactions. This observation leads us to hypothesize that rhizosphere microbes may prioritize engagement with plant-derived resources over forming dense correlative networks with other microbes.

## 4 Discussion

### 4.1 Rainfall pulse theory drives rapid microbial dormancy-activation switch in alpine soil

Rainfall pulses represent a hydrological disturbance in semi-arid alpine ecosystems, and the rainfall pulse theory posits that episodic precipitation triggers abrupt soil rewetting, awakening dormant microbial taxa and inducing rapid metabolic activation within hours (Schimel et al., 2007; Chen et al., 2025). This study provides snapshot evidence that heavy rainfall (12 h accumulation 15.0–29.9 mm) drove immediate microbial restructuring in the Qinghai Lake Basin, which is highly consistent with the findings of Chen et al. (2025) in the alpine sandy ecosystem of Qinghai Lake Bird Island: both studies confirmed that rainfall significantly reduced bacterial alpha diversity and triggered taxonomic and functional shifts within 2 h after rain cessation.

In dry pre-rainfall soils, most microorganisms enter a dormant state to resist drought stress, with reduced metabolic activity and depressed cell division (Manzoni et al., 2012; Chen et al., 2025). Upon rewetting, water infiltration rapidly replenishes soil moisture, dissolves osmolytes, and restores cellular turgor, thereby awakening dormant microbes and initiating rapid substrate utilization (Iqbal et al., 2025; Schimel et al., 2007). Our data showed that post-rainfall microbial reads decreased from ∼40% to ∼25%, which was attributed to the plant DNA dilution effect rather than microbial death, consistent with the observation in Chen et al. (2025) that rainfall reduced microbial relative abundance but did not eliminate core taxa. At the phylum level, Actinomycetota maintained high stability pre- and post-rainfall, identical to the pattern reported by Chen et al. (2025), owing to their thick cell walls and spore-forming traits that confer strong drought resistance and rapid reactivation capacity (Solans et al., 2021). In contrast, Proteobacteria declined sharply in the rhizosphere after rainfall, which was also observed in the Bird Island sandy ecosystem, reflecting their sensitivity to moisture-induced oxygen limitation and root exudate dilution (Chen et al., 2025).

These results collectively validate the rainfall pulse theory in alpine grasslands: short-term heavy rainfall acts as a “metabolic switch” that transitions microbial communities from drought dormancy to active growth, with consistent response patterns across different microhabitats (sandy dunes vs. wetland meadow) of the Qinghai Lake Basin (Chen et al., 2025; Liu et al., 2023).

### 4.2 Rhizosphere carbon flux mediates compartment-specific microbial functional adaptation

The rhizosphere is a hotspot of root carbon flux, where root exudates (sugars, organic acids, amino acids) fuel microbial growth and shape niche differentiation (Trivedi et al., 2020; Hu et al., 2018). Pre-rainfall, the rhizosphere maintained higher total carbon (TC) and total nitrogen (TN) than bulk soil, forming a carbon-rich microenvironment that enriched Proteobacteria—a copiotrophic phylum specialized in labile carbon utilization (Chen et al., 2025). After rainfall, leaching and microbial consumption reduced rhizosphere TC and TN, while bulk soil nutrients increased, indicating rainfall-driven carbon and nitrogen translocation from rhizosphere to bulk soil, a phenomenon also reported in alpine grasslands by Liu et al. (2023).

Metabolically, the rhizosphere microbiome shifted from a pre-rain “carbon-oriented metabolic state” (branched-chain amino acid biosynthesis, porphyrin metabolism) to post-rain increased relative abundance of genes involved in central carbon pathways (TCA cycle, glycolysis) and amino acid degradation, which is highly consistent with Chen et al.’s (2025) finding that rhizosphere microbes transition from carbohydrate catabolism to anaerobic energy production after rainfall. This functional shift reflects rhizosphere carbon flux reconfiguration: rainfall stimulates root exudation and organic matter mineralization, increasing labile carbon availability and driving microbes to prioritize energy metabolism for rapid growth (Brokate et al., 2024; Chen et al., 2025). Specifically, this shift was associated with increased relative abundance of genes involved in central carbon metabolism, which is consistent with the KEGG pathway analysis results showing enrichment of central carbon metabolic pathways (e.g., TCA cycle, Glycolysis/gluconeogenesis) in post-rainfall rhizosphere soils.

In contrast, bulk soil microbes shifted to starch and sucrose metabolism after rainfall, relying on dissolved organic carbon leached from the rhizosphere rather than root-derived fresh carbon (Chen et al., 2025). This compartmentalized functional divergence—rhizosphere focusing on plant-microbe nutrient exchange and bulk soil on exogenous carbon utilization—highlights the core role of rhizosphere carbon flux in mediating microbial responses to rainfall pulses, which is a conserved mechanism in alpine ecosystems (Wan et al., 2024; Chen et al., 2025).

### 4.3 Nutrient limitation in alpine ecosystems modulates microbial community assembly

Alpine grasslands are characterized by strong nitrogen and phosphorus limitation, low temperatures, and slow nutrient mineralization, making rainfall-induced nutrient mobilization a key driver of microbial assembly (Liu et al., 2023; Chen et al., 2025). Pre-rainfall, drought inhibits nutrient mineralization, resulting in low bioavailable nitrogen and carbon, which enriches drought-tolerant taxa (e.g., *Streptomyces*, *Sphingomonas*) that degrade recalcitrant organic matter (Chen et al., 2025). After rainfall, increased moisture accelerates mineralization, releasing labile nutrients and favoring copiotrophic taxa such as *Paraburkholderia*, *Azospirillum*, and *Methylobacterium*—functional groups associated with nitrogen fixation and plant growth promotion (Liu et al., 2024; Chen et al., 2025).

Notably, our study and Chen et al. (2025) both found that rhizosphere microbial communities were more stable than bulk soil under rainfall disturbance, as root exudates buffer nutrient fluctuations and reduce environmental filtering (Trivedi et al., 2020; Chen et al., 2025). Bulk soil microbes were strongly correlated with soil moisture and total carbon, while rhizosphere microbes were less affected by abiotic physicochemical properties, consistent with the “rhizosphere buffering effect” proposed by Chen et al. (2025). This pattern confirms that in nutrient-limited alpine ecosystems, plant-microbe interactions alleviate nutrient stress and enhance microbial resilience to rainfall pulses (Wan et al., 2024; Chen et al., 2025).

### 4.4 Methodological considerations and key limitations

Previous investigations into rainfall pulses in temperate and alpine grasslands have consistently demonstrated that rewetting triggers rapid microbial activation, reduces alpha diversity, and induces a functional shift from drought adaptation to nutrient cycling (Schimel et al., 2007; Yang et al., 2021; Liu et al., 2023), with Yang et al. (2021) reporting that increased precipitation decreased bacterial diversity in a meadow steppe and Liu et al. (2023) finding that rainfall enhanced microbial functional genes associated with carbon and nitrogen cycling in alpine grasslands of northern Tibet—results that align with our observations. Our study extends these findings through a direct comparison with Chen et al. (2025) conducted in the same Qinghai Lake region but in a distinct microhabitat: both studies confirmed that rainfall reduces bacterial alpha diversity, maintains the stability of *Actinobacteria*, decreases *Proteobacteria* in the rhizosphere, and is associated with shifts in microbial functional gene profiles from carbon catabolism toward energy and nutrient metabolism; However, in the sandy Bird Island ecosystem (Chen et al., 2025), rainfall drove a transition from drought-resistant taxa (e.g., *Geobacter*, *Pseudomonas*) to moisture-adapted genera (e.g., *Azospirillum*, *Methylobacterium*), whereas in the Sanjiaocheng wetland meadow (this study), rainfall enriched *Paraburkholderia* and *Nocardioides*, underscoring habitat-specific microbial adaptation. Collectively, these comparisons reveal that rainfall pulse-induced microbial dormancy–activation, taxonomic restructuring, and functional reprogramming represent general mechanisms in alpine grasslands, whereas the specific responses of microbial taxa are modulated by soil texture, vegetation type, and nutrient availability (Chen et al., 2025; Wan et al., 2024).

## 5 Conclusion

In conclusion, our snapshot comparison between pre-rainfall and immediate post-rainfall conditions reveals consistent temporal differences in microbial community structure and function between rhizosphere and bulk soil compartments in the alpine grassland of the Qinghai Lake Basin. Rainfall coincided with reduced microbial alpha diversity and increased community evenness in the rhizosphere, whereas bulk soil diversity remained relatively stable. Functionally, the rhizosphere microbiome shifted from a pre-rain “carbon-oriented” metabolic state toward higher relative abundance of genes involved in central carbon pathways, amino acid degradation, and chemotaxis-related functions. Meanwhile, Paraburkholderia was significantly enriched in this compartment; however, its specific functional role in nitrogen cycling still requires verification at the gene level. In contrast, bulk soil communities shifted toward starch and sucrose metabolism and bacterial secretion systems. Co-occurrence network analysis further showed that microbial interactions were simplified, and coupling between microbial communities and soil physicochemical properties was weakened in conjunction with rainfall. These patterns suggest that moisture-mediated resource availability is associated with niche-dependent restructuring of soil microbiomes, highlighting distinct ecological strategies in rhizosphere versus bulk soil compartments immediately following rainfall pulses. Notably, these interpretations are based on two static time points and represent snapshot associations rather than fully resolved short-term dynamics or definitive causal relationships. Diel cycles, short-term temperature shifts, or other transient factors may have contributed to the observed changes. Within these limitations, this study improves our understanding of how soil microbiomes respond to hydrological pulses in vulnerable high-altitude ecosystems.

## Data Availability

The datasets presented in this study can be found in online repositories. The names of the repository/repositories and accession number(s) can be found in the article/Supplementary material.
